# MicroRNA-33a-5p suppresses growth of osteosarcoma cells and is downregulated in human osteosarcoma

**DOI:** 10.3892/ol.2015.3503

**Published:** 2015-07-17

**Authors:** JUFENG ZHANG, DAPING WANG, JIANYI XIONG, LEI CHEN, JIANGHONG HUANG

**Affiliations:** 1 Department of Biological Sciences, School of Life Science, Guangdong Pharmaceutical University, Guangzhou, Guangdong 510006, P.R. China; 2 Department of Orthopaedics, Shenzhen Second People's Hospital, Shenzhen, Guangdong 518039, P.R. China; 3 Shenzhen Key Laboratory for Tissue Engineering, Shenzhen, Guangdong 518039, P.R. China

**Keywords:** osteosarcoma, microRNA-33a-5p, tumor suppressor gene

## Abstract

A body of evidence has indicated that microRNAs (miRNAs) may have significant roles in cancer. Aberrant expression of miRNAs has frequently been observed in various human malignancies, including osteosarcoma (OS). However, the roles of miRNAs in OS remain poorly understood. In the present study, high-throughput deep sequencing was performed to screen for deregulated miRNAs in OS. Screening identified 310 miRNAs which were significantly overexpressed and 41 miRNAs which were significantly downregulated (>2-fold) in OS samples, compared with adjacent non-tumor bone tissues. Among these miRNAs, miR-33a-5p was notably downregulated. TaqMan reverse transcription-polymerase chain reaction analysis further verified that miR-33a-5p expression was significantly reduced in a large cohort of human OS samples. Enhancing miR-33a-5p expression via transfection with miR-33a-5p precursor significantly inhibited OS cell growth, suggesting potential antitumor properties of miR-33a-5p. The results of the present study provide novel insights into the miRNAs involved in OS, and suggest that miR-33a-5p may function as a tumor suppressor in OS. Therefore, miR-33a-5p may be able to serve as a diagnostic and therapeutic target for OS treatment.

## Introduction

Osteosarcoma (OS) is the most common primary bone malignancy, which mainly occurs in children and young adolescents (1–3). OS is a complex tumor, characterized by numerous chromosomal alterations and extensive gene mutations (4,5). Numerous molecular studies of OS have been undertaken in recent years, the results of which have provided insight into the molecular pathogenesis of OS (4–9). However, the fundamental molecular mechanisms underlying the occurrence and development of this sarcoma remain to be fully elucidated. Despite significant advances in the development of multimodality treatments comprising wide tumor excision with aggressive adjuvant chemotherapy, the prognosis of patients with recurrent or metastatic OS remains poor (10,11). Therefore, the identification of novel molecular biomarkers to facilitate the early diagnosis and therapy of OS is required, in order to improve the clinical outcome of patients with OS (12).

MicroRNAs (miRNAs) are small non-coding RNAs of ~22 nucleotides, which have emerged as a major class of regulatory genes in animals and plants (13,14). miRNAs are estimated to regulate >30% of the human genome, and are therefore involved in diverse functions, including development, cell differentiation, regulation of the cell cycle and apoptosis (15). A number of studies have demonstrated that miRNA alterations are involved in the development of human cancer (16,17). miRNA genes were frequently demonstrated to be located in cancer-associated genomic regions or fragile sites, suggesting that miRNAs in the genome may be extensively involved in cancer (18). In addition, miRNAs may function as oncogenes or tumor suppressors depending on the nature of their targets (19–21).

Overexpressed miRNAs in various types of cancer, including the miR-17-92 cluster, which comprises 7 miRNAs and is located in intron 3 of the C13orf25 gene at 13q31.3, have been reported to function as oncogenes and accelerate tumor development (22,23). Tumor-suppressive miRNAs, for example the let-7 family, are located in fragile regions of the human genome, and their loss is indicative of poor prognosis in various types of human cancer (24). Accumulating evidence suggests that miRNA expression profiling may be used in the classification of human cancers, indicating the potential of miRNAs as a novel diagnostic and prognostic tools for various types of cancer (25).

Several recent studies have identified a number of dysregulated miRNAs in OS (26–29). Maire *et al* (30) performed miRNA expression profiling of seven OS samples and identified several aberrantly expressed miRNAs. Lulla *et al* (29) identified 22 differentially expressed miRNAs in OS tumor samples, compared with normal osteoblasts. However, given the number of aberrant miRNAs identified thus far, it was suggested that there may be additional miRNAs involved in OS, which remain to be identified. Recent advances in high-throughput deep sequencing have markedly increased the speed of the search for cancer-associated miRNAs (31,32). High-throughput deep sequencing indicates the expression levels of each miRNA in the miRNome, and is thus one of the most effective and accurate approaches for evaluation of global miRNA expression levels (33,34). To the best of our knowledge, deep sequencing of the miRNome associated with OS has not previously been performed, and such comprehensive analysis may provide insight into the molecular mechanisms of OS.

The present study aimed to comprehend current literature by complete profiling of OS miRNA expression patterns, and further evaluating significantly differentially expressed miRNAs and their effects on human OS cells *in vitro*.

## Materials and methods

### 

#### Patients and samples

The primary OS samples and matched noncancerous bone tissue samples used for the high-throughput deep sequencing experiments were obtained from two patients with OS undergoing surgical resection at Shenzhen Second People's Hospital (Shenzhen, China). Collected samples were flash frozen in liquid nitrogen following surgery. All patients were informed about the aims of the specimen collection and provided written informed consent. For the reverse transcription-quantitative polymerase chain reaction (RT-qPCR) verification, 32 OS samples were obtained from an archive of formalin-fixed, paraffin-embedded (FFPE) diagnostic tissues in the Pathology Department at Shenzhen Second People's Hospital, collected between 1990 and 2013. All tumor samples were high-grade OS of stage IIA or IIB, according to the Enneking system (35). The mean age of the patients was 21 years (range, 18–36 years), and 59% were male. All diagnoses were confirmed by an experienced pathologist. Specimens from 36 normal muscles of patients who had undergone orthopedic surgery were collected and immediately stored in liquid nitrogen prior to use as the negative controls. The study was approved by the ethics committee of Shenzhen Second People's Hospital.

#### Identification of differentially expressed miRNAs

High-throughput deep sequencing was performed using the Illumina Cluster Station and Genome Analyzer II (Illumina Inc., San Diego, CA, USA). Small RNA library construction, sequencing and bioinformatics analysis was conducted as previously described (36,37). The miRNA expression levels were compared between OS samples and paired normal bone tissues to detect the differentially expressed miRNAs. The expression levels of miRNAs in two samples were first normalized to obtain the expression of transcripts per million, and the fold-change and P-values were then calculated from the normalized expression level. In general, if the adjusted P-values were <0.01 based on the Benjamini and Hochberg multiple testing correction (38) and there was a ≥2-fold change (OS samples/paired normal bone tissues) in the normalized expression, the miRNA was considered to be significantly differentially expressed.

#### Total RNA extraction and reverse transcription-quantitative polymerase chain reaction (RT-qPCR) of miRNAs

FFPE samples were cut into 10-µm sections. Total RNA was isolated from FFPE samples using the Qiagen RNeasy FFPE protocol (Qiagen, Inc., Valencia, CA, USA). For surgical resection specimens, total RNA was extracted using the mirVana miRNA isolation kit (Ambion, Austin, TX, USA) according to the manufacturer's instructions.

RT-qPCR analysis of mature miR-33a-5p was performed in triplicate using the TaqMan MicroRNA assay kit (Ambion) according to the manufacturer's instructions. The RT reaction mixture was comprised of 10 ng total RNA, 1 mM deoxynucleotide triphosphates, 50 U Multiscribe Reverse Transcriptase, 1.5 µl 10X RT buffer, 0.188 µl RNase inhibitor and 3 µl 5X TaqMan MicroRNA RT primer for each reaction (15 µl). The RT reaction was performed under the following conditions: 16°C for 30 min; 42°C for 30 min and 85°C for 5 min, prior to holding at 4°C. Following RT, the complementary DNA products of the RT reaction were diluted 15 times. PCR was conducted using 1.33 µl of the diluted product in 20 µl PCR reaction mixture, comprising 1 µl TaqMan MicroRNA Assay and 10 µl TaqMan Universal PCR Master mix. Subsequently, amplification was performed under the following conditions: 95°C for 10 min, followed by 40 cycles of 95°C for 15 s and 60°C for 60 s. Relative expression was calculated using the comparative C(T) method (39) and normalized to the expression of RNU6B (Ambion).

miRNAs identified by high-throughput deep sequencing were validated using the miScript PCR System (Qiagen, Inc., Gaithersburg, MD, USA) according to the manufacturer's instructions. The RT reaction mixtures with the miScript II RT kit (Qiagen) contained 1 µg total RNA, 4 µl 5X miScript HiSpec buffer, 2 µl 10X miScript nucleics mix and 2 µl miScript reverse transcriptase mix for each reaction (20 µl). RT was performed under the following conditions: 37°C for 60 min, followed by 95°C for 5 min. Subsequently, the cDNA products of the RT reaction were diluted 15 times. PCR was performed with 1.5 µl of the diluted products in 20 µl PCR reaction mixture containing 10 µl 2X QuantiTect SYBR Green PCR master mix, 2 µl 10X miScript universal primer, 2 µl 10X miScript primer assay. Amplification was performed under the following conditions: 95°C for 15 min, followed by 40 cycles at 94°C for 15 sec, 55°C for 30 sec and 70°C for 30 sec. All reactions were performed in triplicate. Relative expression was calculated using the comparative C(T) method and normalized to the expression of RNU6B.

#### Cell culture

The human U2-OS and MG-63 OS cell lines were purchased from the Type Culture Collection of the Chinese Academy of Sciences (Shanghai, China). U2-OS and MG-63 cells were cultured in McCoy's 5A media (modified with tricine; (Gibco Life Technologies, Grand Island, NY, USA) and minimum essential medium supplemented with 10% fetal bovine serum (Gibco), respectively. Cells were incubated at 37°C in a 5% CO_2_ atmosphere.

#### miR-33a-5p precursor transfection

The miR-33a-5p precursor and random sequence CY3-labeled miR-Scramble were synthesized by Ambion. U2-OS and MG-63 cells were counted and plated at a density of 4×10^5^ cells/well in 6-well plates for overnight incubation prior to transfection with 100 nM miR-33a-5p precursor or miR-Scramble using Lipofectamine® 2000 (Invitrogen Life Technologies, CA, USA) according to the manufacturer's instructions. Transfection efficiency was estimated by CY3-labeled miR-Scramble using a fluorescence microscope (Axio Observer A1; Zeiss, Jena, Germany).

#### Cell proliferation assays

The effect of miR-33a-5p on cell proliferation was measured by WST-1 assay. Cells were counted and plated at a density of 3×10^3^ cells/well in 96-well plates in triplicates. Cell viability was determined at 24, 48 and 72 h post-transfection. Spectrophotometry (Beckman DU spectrophotometer, Beckman-Coulter, Brea, CA, USA) was performed at λ=450 nm and λ ref=630 nm following incubation with 10 µl WST-1 (Roche Diagnostics, New York, NY, USA) for 2 h. Cell proliferation was evaluated using a colony formation assay. Briefly, cells were seeded in six-well plates (0.5×10^3^ cells/well) and cultured for two weeks. Colonies were fixed with methanol for 10 min and stained with 1% crystal violet (Sigma-Aldrich, St. Louis, MO, USA) for 1 min. Visible colonies (defined as containing >50 cells) in 10 random fields were manually counted. Each cell group was measured in triplicate.

#### Statistical analysis

miR-33a-5p expression in OS samples and normal bone or muscle tissues were compared using the Mann-Whitney U test. Correlation was evaluated using Pearson's Correlation Coefficient. A comparison of means among two or more groups was performed using one-way analysis of variance or Student's t-test. All numerical data are expressed as the mean ± standard deviation. P<0.05 was considered to indicate a statistically significant difference. Statistical analyses were performed using GraphPad Prism 5.0 (GraphPad Software, Inc., La Jolla, CA, USA) and SPSS software version 11 (SPSS, Inc., Chicago, IL, USA).

## Results

### 

#### miRNAs differentially expressed in OS and normal bone tissues

**Figure 1.**
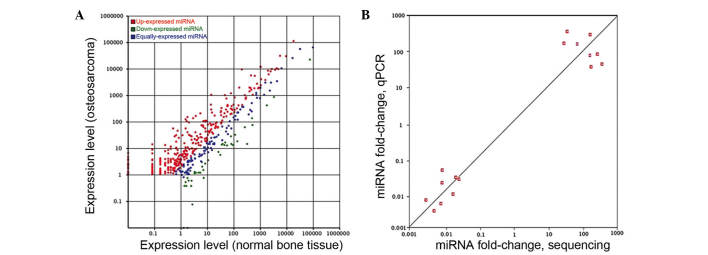
Evaluation of miRNA expression levels by deep sequencing and validation with RT-qPCR. (A) Scatter plot indicating the expression levels of known miRNAs in osteosarcoma and adjacent normal bone tissues. Blue, miRNAs equally expressed in osteosarcoma and normal bone tissue; red, miRNAs upregulated in osteosarcoma compared with those of normal bone tissue (adjusted P<0.05); green, miRNAs downregulated in osteosarcoma compared with those of normal bone tissue (adjusted P<0.05). (B) Validation of eight selected up- and downregulated miRNAs indicated that the results from deep sequencing correlated with the RT-qPCR results. miRNA, microRNA; RT-qPCR, reverse transcription-quantitative polymerase chain reaction.

To investigate the expression profiles of miRNAs in OS and adjacent normal bone tissues, high-throughput deep sequencing was used to compare their expression levels. High-throughput deep sequencing revealed a series of miRNAs with altered expression in OS tissues: 310 miRNAs were significantly overexpressed and 41 miRNAs were significantly downregulated (>2-fold; adjusted P<0.05), compared with those of normal tissues (Fig. 1A). Among these differentially expressed miRNAs, a total of 47 miRNAs with >32-fold elevated expression levels and 17 miRNAs with expression levels reduced >4-fold were identified (Table I).

To further validate these differentially expressed miRNAs, eight miRNAs identified by high-throughput deep sequencing were re-examined by RT-qPCR. The eight miRNAs selected included the most upregulated miRNAs (miR-512-3p, miR-377-5p, miR-433-3p and miR-1323) and most downregulated miRNAs (miR-33a-5p, miR-551b-3p, miR-3613-5p and miR-144-3p) in OS. As illustrated in Fig. 1B, the Illumina deep sequencing data correlated with the RT-qPCR results (r=0.805; P<0.001), indicating the reliability of sequencing-based expression analysis.

#### miR-33a-5p expression is decreased in paraffin-embedded OS samples

**Figure 2.**
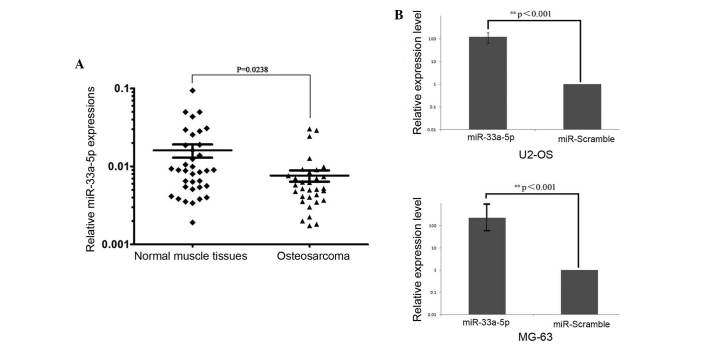
miR-33a-5p expression is decreased in OS cells and tissues. (A) Relative expression of miR-33a-5p was evaluated by TaqMan (RT-qPCR). Human normal muscle RNAs were used as controls. miR-33a-5p expression was downregulated in OS tissues. (B) Confirmation of overexpression of miR-33a-5p in transfected cell lines by real-time PCR. OS cell lines U-2OS and MG-63 were transfected with miR-33a-5p precursor and miR-Scramble control, and the relative expression of miR-33a-5p was assessed by RT-qPCR. Values are presented as the mean ± standard deviation. miR, microRNA; RT-qPCR, reverse transcription-quantitative polymerase chain reaction; OS, osteosarcoma.

Subsequently, miR-33a-5p was further analyzed, as this was the most downregulated miRNA identified in the OS samples. To verify the expression levels of miR-33a in OS, miR-33a-5p expression levels were determined in 32 paraffin-embedded human OS samples and 36 normal muscle tissues by TaqMan RT-qPCR. As shown in Fig. 2A, miR-33a-5p expression was significantly downregulated in paraffin-embedded OS tissues, compared with that of normal muscle tissue (P=0.0238). These results suggested that miR-33a-5p may have a role in the pathogenesis of OS.

#### miR-33a-5p inhibits OS cell proliferation

To investigate the functional role of miR-33a-5p in OS, human U2-OS and MG-63 OS cell lines were transfected with 100 nM chemically synthesized miR-33a-5p precursor, which mimics endogenous mature miR-33a-5p function. Cells transfected with 100 nM miR-Scramble (scrambled oligonucleotides) were used as the control. Transfection efficiency of miRNA in these two cell lines was estimated by CY3-labeled miR-Scramble (>80%; data not show). Additionally, 24 h post transfection, miR-33a-5p expression levels were evaluated by RT-qPCR. The results demonstrated that miR-33a-5p mimic enhanced miR-33a-5p expression by ~152-fold (P<0.001) in U2-OS cells and ~341-fold in MG-63 cells (P<0.001), compared with the scramble-transfected group (Fig. 2B). These results indicated that the miR-33a-5p precursor was able to effectively increase miR-33a-5p expression in U2-OS and MG-63 cells.

**Figure 3.**
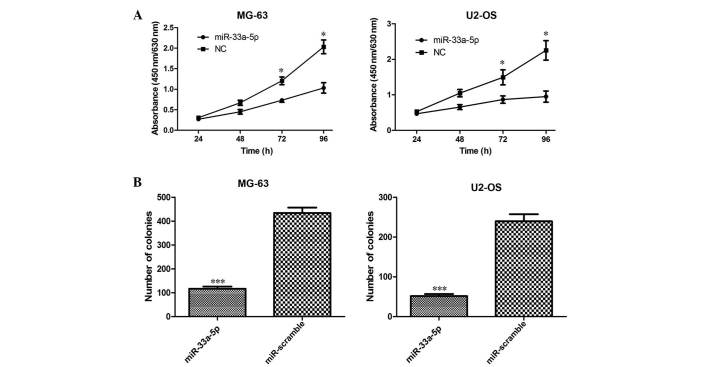
Osteosarcoma cancer cell growth is suppressed by miR-33a-5p precursor overexpression. (A) WST-1 assay indicated that MG-63 and U2-OS cells transfected with miR-33a-5p precusor grew more slowly than cells transfected with miR-Scramble. (B) Colony formation assay of miR-33a-5p precusor and miR-Scramble-transfected MG-63 and U2-OS cells indicated that miR-33a-5p supressed colony formation. All data are presented as the mean ± standard deviation. *P<0.05; ***P<0.001 vs. miR-Scramble-transfected cells. miR, microRNA; NC, normal control.

Following 48, 72 and 96 h of incubation, U2-OS and MG-63 cells overexpressing miR-33a-5p exhibited decreased cell proliferation, as compared with miR-Scramble-transfected cells, respectively (P<0.05; Fig. 3A). Consistent with these results, in the WST-1 assay, cells transfected with miR-33a-5p demonstrated formation of significantly fewer colonies than those of cells transfected with the miR-Scramble (P<0.001; Fig. 3B).

## Discussion

In the present study, miRNAs that were up- or downregulated in OS, as compared with matched noncancerous bone tissues, were detected through high-throughput deep sequencing. The results demonstrated that the expression levels of 310 miRNAs were increased and 41 miRNAs were decreased in the OS tissues. Among these, miR-512-3p, miR-377-5p, miR-433-3p and miR-1323 were the greatest upregulated miRNAs, whereas miR-33a-5p, miR-551b-3p, miR-3613-5p and miR-144-3p were the most decreased miRNAs in OS. These miRNAs were re-examined by RT-qPCR analysis and the results correlated with those of the sequencing analysis. Specifically, miR-33a-5p was decreased most in OS, which suggested that miR-33a-5p may have a role in the pathogenesis of OS.

To the best of our knowledge, the role of miR-33a-5p in OS has not previously been reported. miR-33a-5p has recently emerged as a key regulator of metabolism, and was shown to regulate cholesterol and lipid metabolism (40,41). miR-33a is downregulated in lung cancer cells and functions as a potent tumor suppressor, which decreases osteolytic bone metastasis via suppression of parathyroid hormone-related protein (42). miR-33a also functions as a tumor suppressor miRNA through its capacity to downregulate the expression of oncogenic kinase Pim-1 in K562 lymphoma and colon carcinoma (43,44). miR-33 family members have been associated with modulation of the expression of various genes involved in cell cycle regulation and proliferation (45,46). miR-33 decreases cellular proliferation and cell cycle progression via inhibition of cyclin-dependent kinase 6 and cyclin D1 (45,46). In the present study, miR-33a-5p was demonstrated to be downregulated in OS, while overexpression of miR-33a-5p by transfection, significantly attenuated OS cell growth *in vitro*.

The aberrant expression of miRNAs may occur via a number of mechanisms, for example by deletion in fragile regions of the genome containing cancer-suppressing miRNAs, as a result of inherent or spontaneous mutations in miRNA genes or following methylation of miRNA promoters (47–50). However, the mechanism by which miR-33a-5p is downregulated in OS remains to be elucidated.

In conclusion, the results of the present study demonstrated that multiple miRNAs are aberrantly expressed in human OS. Among these miRNAs, miR-33a-5p is significantly downregulated in the majority of OS tissues. miR-33a-5p demonstrated tumor suppressive abilities *in vitro* by inhibiting OS cell proliferation, which suggested that miR-33a-5p may have a tumor suppressor function in human OS. These results provide support for the rescue of miR-33a-5p expression via gene therapy, and demonstrated the potential use of miR-33a-5p as diagnostic marker or therapeutic tool for the treatment of human OS.

## Figures and Tables

**Table I.** Significantly differentially expressed known miRNAs in osteosarcoma.

miRNA ID	Log_2_, fold-change^a^	Adjusted P-value
Upregulated		
hsa-miR-512-3p	9.39	2.80×10^−62^
hsa-miR-377-5p	8.90	1.95×10^−9^
hsa-miR-433-3p	8.60	1.54×10^−5^
hsa-miR-1323	8.45	4.72×10^−5^
hsa-miR-337-3p	8.11	5.30×10^−22^
hsa-miR-485-3p	8.07	8.00×10^−56^
hsa-miR-6503-5p	8.07	8.08×10^−7^
hsa-miR-656-3p	7.99	<0
hsa-miR-411-3p	7.94	6.82×10^−8^
hsa-miR-494-3p	7.85	1.98×10^−4^
hsa-miR-4709-5p	7.81	7.79×10^−4^
hsa-miR-508-3p	7.76	1.23×10^−6^
hsa-miR-187-3p	7.54	6.42×10^−12^
hsa-miR-370-5p	7.54	4.12×10^−9^
hsa-miR-105-3p	7.42	1.08×10^−105^
hsa-miR-873-3p	7.37	3.22×10^−4^
hsa-miR-1185-5p	7.07	1.36×10^−51^
hsa-miR-541-3p	7.07	7.67×10^−6^
hsa-miR-885-5p	7.07	3.50×10^−16^
hsa-miR-329-5p	6.99	7.26×10^−4^
hsa-miR-337-5p	6.99	1.84×10^−3^
hsa-miR-219a-1-3p	6.90	2.34×10^−13^
hsa-miR-329-3p	6.90	<0
hsa-miR-134-3p	6.81	<0
hsa-miR-134-5p	6.80	1.83×10^−22^
hsa-miR-654-5p	6.77	<0
hsa-miR-758-3p	6.77	1.44×10^−5^
hsa-miR-487b-3p	6.72	2.48×10^−6^
hsa-miR-20b-3p	6.71	3.18×10^−292^
hsa-miR-380-3p	6.71	3.27×10^−5^
hsa-miR-654-3p	6.42	4.30×10^−31^
hsa-miR-432-5p	6.35	1.67×10^−16^
hsa-miR-105-5p	6.25	3.64×10^−22^
hsa-miR-409-3p	6.21	2.26×10^−3^
hsa-miR-1269b	5.97	9.01×10^−4^
hsa-miR-493-3p	5.93	5.29×10^−97^
hsa-miR-431-3p	5.73	<0
hsa-miR-127-3p	5.57	3.27×10^−11^
hsa-miR-409-5p	5.56	5.01×10^−3^
hsa-miR-370-3p	5.54	5.75×10^−7^
hsa-miR-767-5p	5.54	2.20×10^−7^
hsa-miR-410-3p	5.54	9.27×10^−3^
hsa-miR-493-5p	5.51	<0
hsa-miR-487a-3p	5.50	1.24×10^−8^
hsa-miR-520a-3p	5.41	4.47×10^−10^
hsa-miR-381-3p	5.24	4.87×10^−14^
hsa-miR-149-5p	5.22	1.42×10^−3^
Downregulated		
hsa-miR-33a-5p	−7.46	<0
hsa-miR-551b-3p	−6.98	6.63×10^−5^
hsa-miR-3613-5p	−5.17	1.53×10^−3^
hsa-miR-144-3p	−4.47	<0
hsa-miR-190a-5p	−3.28	6.73×10^−83^
hsa-miR-335-5p	−2.90	1.59×10^−9^
hsa-miR-144-5p	−2.75	9.27×10^−13^
hsa-miR-224-3p	−2.71	1.72×10^−140^
hsa-miR-193a-3p	−2.46	1.63×10^−55^
hsa-miR-19a-3p	−2.46	4.56×10^−6^
hsa-miR-33b-5p	−2.26	<0
hsa-miR-452-3p	−2.17	9.84×10^−289^
hsa-miR-29c-3p	−2.12	1.43×10^−18^
hsa-miR-101-3p	−2.11	<0
hsa-miR-2467-5p	−2.10	5.55×10^−10^
hsa-miR-378a-5p	−2.08	1.13×10^−5^
hsa-miR-145-5p	−2.04	1.22×10^−7^

a fold change = (osteosarcoma/normal bone tissue). miR/miRNA, microRNA; ID, identification; hsa, *Homo sapiens*.
